# High School Students Residing in Educational Public Institutions: Health-Risk Behaviors

**DOI:** 10.1371/journal.pone.0161652

**Published:** 2016-08-25

**Authors:** Priscilla Rayanne e Silva Noll, Nusa de Almeida Silveira, Matias Noll, Patrícia de Sá Barros

**Affiliations:** 1 Graduate Program in Public Health, Federal University of Goiás, Goiânia, Goiás, Brazil; 2 Goiano Federal Institute—Ceres Campus, Ceres, Goiás, Brazil; National Institute of Health, ITALY

## Abstract

Although several health-risk behaviors of adolescents have been described in the literature, data of high school students who reside at educational institutions in developing countries are scarce. This study aimed to describe behaviors associated with health risks among high school students who reside at an educational public institution and to associate these variables with the length of stay at the institution. This cross-sectional study was conducted during the year 2015 and included 122 students aged 14–19 years at a federal educational institution in the Midwest of Brazil; students were divided into residents of <8 months and those of >20 months. Information concerning the family socioeconomic status and anthropometric, dietary and behavioral profiles was investigated. Despite being physically active, students exhibited risk-associated behaviors such as cigarette and alcohol use and risky sexual behaviors that were exacerbated by fragile socioeconomic conditions and distance from family. A longer time in residence at the institution was associated with an older age (p ≤ 0.001), adequate body mass index (BMI; p = 0.02), nutritional knowledge (p = 0.01), and less doses of alcohol consumption (p ≤ 0.01) compared with those with shorter times in residence. In conclusion, the students exhibited different health-risk behaviors, and a longer institutional residence time, compared with a shorter time, was found to associate with the reduction of health-risk behaviors.

## Introduction

During adolescence, an intense transition phase associated with maturation, a high prevalence of various health-risk behaviors such as cigarette, alcohol, and illicit drug use, poor eating habits, insufficient physical activity, and risky sexual behavior, all of which contribute to adulthood behaviors, is observed among students [1–3]. Health-risk conditions and socioeconomic inequalities, which are intensified in socioeconomically weak environments, remain neglected issues with respect to youth [4–7].

The right to housing is conferred upon teenagers who attend high school at some educational institutions; this is considered a subsidy policy and exists in the context of other public programs and policies for this age group [8,9]. According to the literature, such housing benefits are associated with student retention and better school performance [8,10,11]. In all states of Brazil, the Federal Professional Scientific and Technological Education Network provides professional and technological education in different teaching modalities, thus encouraging the integration and verticalization of basic education to professional and higher education and strengthening clusters as well as social and local cultures [12]. On average, this network offers 500,000 annual vacancies for high school students, and approximately 15% of these vacancies are linked with a housing benefit in an attempt to reduce socioeconomic disparities [9], as observed in other countries.

Studies of college students who reside at educational institutions have described health-risk behaviors often associated with distance from family; similar behaviors were observed in one of the few studies involving adolescent students in Israel [13]. The use of alcohol, cigarettes, and marijuana, risky sexual behaviors, insufficient physical activity, and poor eating habits were mentioned most frequently [14–19], and these were found to associate with an increased time of residence in institutional housing [1,14,17].

Our investigation was motivated by the scarcity of studies of high school students who reside at educational institutions [8,20] in developing countries (e.g., Latin America), as well as the importance of knowledge about health risk factors [3,21] in terms of the evaluation of public programs and policies aimed at planning health promotion and disease prevention activities. In light of this gap in the literature, which exists despite the presence of institutions with housing, we aimed to (1) describe the profiles of high school students who reside at an educational institution with respect to health-risk behaviors and (2) correlate these variables with the length of stay at the institution.

## Materials and Methods

### Design, population and sample

This cross-sectional study was conducted in 2015 and involved students residing at a federal public educational institution located in the Midwestern region of Brazil. In this federal institution, vacancies are open annually through public tenders, allowing interested candidates to sign up from several states of Brazil; admission is granted to those who pass the entrance exam. Among the total number of students in the institution (n = 1440), institutional housing is intended for students with lower socioeconomic status, through selection of applicants. All resident students at the institution (n = 134) from seven states of Brazil were invited to participate in the study. The inclusion criteria for this study were as follows: being regularly enrolled in high school; being resident students; being 14–19 years of age; and being without cognitive deficit. As 12 students (8.9%) refused to participate, the final sample comprised 122 students (82% male, n = 100; 18% female, n = 22).

### Measurements

We collected the following information related to adolescent health-risk behaviors [22]: family socioeconomic status, anthropometric profile, eating habits, physical activity, use of cigarettes, alcohol and drugs, and risky sexual behavior.

### Questionnaire

A self-administered questionnaire was developed [23] and validated [24] for use in the National Student Health Survey (*Pesquisa Nacional de Saúde do Escolar*, PENSE), developed by the Brazilian Institute of Geography and Statistics (*Instituto Brasileiro de Geografia e Estatística*) [23]. Our research used the following question blocks: demographic (age, sex, place of origin [urban or rural zone]); socioeconomic (ethnicity/race, education level of legal guardians [elementary school, high school, higher education) and monthly family income relative to the minimum wage in Brazil in 2015 [<1 salary, <R$788.00; 1–3 salaries, R$788–2364.00; >3 salaries, >R$2364.00]; nutrition (consumption of healthy and unhealthy dietary items) and behavioral (physical activity, use of cigarettes, alcohol, and/or illicit drugs, and sexual and reproductive health).

A healthy diet score was obtained by summing of scores of items from the above classifications of markers at five weekly frequency levels (Table 1), ranging from 0 to 44 points [25]. The internal consistency of the score was assessed by correlating the items and by calculating Cronbach's coefficient, which yielded a value of 0.68.

**Table 1:** Points for calculating the healthy diet score.

	Points				
Variable	0	1	2	3	4
**Healthy diet markers**					
Beans	Never/almost never	1-2x/week	3-4x/week	5-6x/week	Every day
Raw salad	Never/almost never	1-2x/week	3-4x/week	5-6x/week	Every day
Cooked vegetables	Never/almost never	1-2x/week	3-4x/week	5-6x/week	Every day
Fresh fruit/juice	Never/almost never	1-2x/week	3-4x/week	5-6x/week	Every day
Milk and dairy products	Never/almost never	1-2x/week	3-4x/week	5-6x/week	Every day
Breakfast	Never/almost never	1-2x/week	3-4x/week	5-6x/week	Every day
**Unhealthy diet markers**					
Fried snacks	Every day	5-6x/week	3-4x/week	1-2x/week	Never/almost never
Sausage	Every day	5-6x/week	3-4x/week	1-2x/week	Never/almost never
Sweet cookies	Every day	5-6x/week	3-4x/week	1-2x/week	Never/almost never
Sweets and desserts	Every day	5-6x/week	3-4x/week	1-2x/week	Never/almost never
Soft drinks and processed juice	Every day	5-6x/week	3-4x/week	1-2x/week	Never/almost never

Regarding physical activity, we assessed the average time in physical activity participation from students’ reports. We considered students who participated in ≥60 minutes daily to be active [26]. The use of substances (cigarettes, alcohol, and illicit drugs) was considered relevant for students who reported having consumed them in the last 30 days [2,27]. We also evaluated the number of doses of alcohol consumed on any occasion. The responses were grouped into three categories (≤1 dose, 2–4 doses, or ≥5 doses). Sexual behavior was assessed according to reports of an active sex life, number of sexual partners, and age at first intercourse. We also verified the use of contraceptives and sexually transmitted disease (STD) prevention methods in addition to previous adherence to guidelines related to sexual and reproductive health in the school environment.

## Anthropometry

The anthropometric assessment consisted of measurements of body mass, height, and waist and neck circumferences. We calculated the body mass index (BMI), which is considered predictive of body fat, relative to age using the z-score: normal weight (-2 SD <BMI z-score <1 SD), overweight (1 SD < BMI z-score <2 SD), or obese (BMI Z-score >2 SD), based on the World Health Organization’s Growth Reference [28]. Students were instructed to wear light clothes at the time of data collection and to remain in the indicated proper position [29]. The data collection team was previously trained.

Waist and neck circumferences were measured using a strong, flexible, non-elastic, tape measure with an accuracy of 0.1 cm [29]. Waist circumference was measured at the midpoint between the lower margin of the last palpable rib and the top of the iliac crest, with the subject in a standing position [30]; this was classified as “increased risk of cardiovascular disease” when in the ≥90^th^ percentile, adjusted for age and sex for students until 18 years of age [31], and as “increased risk of cardiovascular disease” (increased and substantially increased) when >94 cm, for male students and >80 cm for female students older than 18 years [31]. Neck circumference was measured in a horizontal plane at the level of the most prominent portion, the thyroid cartilage [32]. Neck circumference was classified as adequate or indicative of overweight, according to specific cut-off values with respect to age and sex [32–34]. All measurements were taken with the subjects standing upright, with the face directed toward, and shoulders relaxed.

### Nutritional knowledge

The Nutritional Knowledge Scale Questionnaire, which was psychometrically translated, adapted and validated from the instrument published by *National Health Interview Survey Cancer Epidemiology* [35], consists of 12 questions divided into 3 parts: 1) 4 questions about the relationship between diet and disease; 2) 7 questions about the fiber and lipid contents of foods; and 3) 1 question about the number of servings of fruits and vegetables that should be eaten. We asked each student to mark only 1 answer to each questions, and each correct answer received a score of 1.0. At the end, each student’s nutritional knowledge was classified as low (0–6 points), moderate (7–10 points), or high (>10 points).

### Statistical analysis

Students were grouped according to those who were freshmen in 2015 (Group 1, <8 months in the educational institution) and those who were freshmen in 2014 (Group 2, >20 months in the institution) for comparisons of variables relative to the time in residence at the institution. Data were analyzed using descriptive statistics and the chi-square association test (bivariate analysis); time at the institution was set as the dependent variable, and demographic, socioeconomic, anthropometric, nutritional and behavioral factors were set as the independent variables. The analyses were performed using the *Statistical Package for the Social Sciences* version 20.0 (SPSS, Inc., Chicago, IL, USA), and the significance level was set at p = 0.05.

### Ethical considerations

This study was conducted in accordance with the Declaration of Helsinki and was approved by the Ethics Committee on Research with Human Beings for the Federal University of Goiás, Brazil (Opinion no. 1.003.926). Students were told that they could leave the study at any time if they chose not to participate in any of the procedures. Prior to participation, the students were asked to read and voluntarily sign the study consent form; the legal guardians of minor participants also read and signed this form.

## Results

Details of the demographic and socioeconomic data are presented in Table 2.

**Table 2:** Demographic and socioeconomic data of the population studied (n = 122).

Demographic and socioeconomic data		n (%)
State of previous residence	Goiás	104 (85.2)
	Tocantins	10 (8.2)
	Mato Grosso	4 (3.3)
	Outros	4 (3.3)
Family income	<1 minimum wage^a^	46 (37.7)
	1–3 minimum wages	63 (51.6)
	>3 minimum wages	13 (10.7)
Skin color	White	26 (21.3)
	Brown	74 (60.7)
	Black	22 (18.0)
	Yellow and/or indigenous	0 (0)

^a^Refers to the minimum wage in Brazil in the year 2015 (R$788.00).

The results demonstrated that age, nutritional knowledge, and BMI were associated with timing of arrival at the institution (Table 3).

**Table 3:** Association between freshmen in 2015 and freshmen up to 2014 with the independent variables.

Independent variables	n (%)	Group 1	Group 2	χ² ^a^
		n (%)	n (%)	
**Demographic**				
*Age (years) (n = 122)*				0.007^b^
14–16	72 (59)	48 (69.6)	24 (45.3)	
17–19	50 (41)	21 (30.4)	29 (54.7)	
*Sex (n = 122)*				0.791
Male	100 (82)	56 (81.2)	44 (83)	
Female	22 (18)	13 (18.8)	9 (17)	
*Origin/Zone (n = 122)*				0.552
Urban	84 (68.9)	46 (66.7)	38 (71.7)	
Rural	38 (31.1)	23 (33.3)	15 (28.3)	
**Socioeconomic**				
*Ethnicity/Race (n = 122)*				0.551
White	26 (21.3)	16 (23.2)	10 (18.9)	
Brown and/or black	96 (78.7)	53 (76.8)	43 (81.1)	
*Family income (n = 122)*				0.928
<1 minimum wage	46 (37.7)	27 (39.1)	19 (35.8)	
1–3 minimum wages	63 (51.6)	35 (50.7)	28 (52.8)	
>3 minimum wages	13 (10.7)	7 (10.2)	6 (11.4)	
*Maternal education (n = 121)*				0.225
Elementary school	54 (44.6)	35 (51.5)	19 (35.8)	
High school	38 (31.4)	19 (27.9)	19 (35.8)	
Higher education	29 (24)	14 (20.6)	15 (28.4)	
*Paternal education (n = 112)*				0.178
Elementary school	62 (55.4)	38 (62.3)	24 (47.1)	
High school	36 (32.1)	18 (29.5)	18 (35.3)	
Higher education	14 (12.5)	5 (8.2)	9 (17,6)	
**Anthropometric**				
*BMI (n = 121)*				0.027^b^
Normal weight	96 (79.3)	50 (72.5)	46 (88.5)	
Overweight	25 (20.7)	19 (27.5)	6 (11.5)	
*Waist circumference (n = 122)* ^*c*^				0,257
Adequate	119 (97.5)	66 (95.7)	53 (100)	
Increased	3 (2.5)	3 (4.3)	0 (0)	
*Neck circumference (n = 122)*				0.631
Adequate	73 (59.8)	40 (58)	33 (62.3)	
Indicative of overweight	49 (40.2)	29 (42)	20 (37.7)	
**Nutritional**				
*Healthy diet score (n = 122)*				0.823
≤30 points	91 (74.6)	52 (75.4)	39 (73.6)	
>30 points	31 (25.4)	17 (24.6)	14 (26.4)	
*Nutritional knowledge (n = 118)*				0.017^b^
Low knowledge	54 (45.8)	37 (55.2)	17 (33.3)	
Moderate to high knowledge	64 (54.2)	30 (44.8)	34 (66.7)	
**Behavioral**				
*Physical activity (n = 122)*				0.346
Active	86 (70.5)	51 (73.9)	35 (66)	
Insufficiently active	36 (29.5)	18 (26.1)	18 (34)	
*Use of cigarettes last month (n = 122)*				0.979
No	106 (86.9)	60 (87)	46 (86.8)	
Yes	16 (13.1)	9 (13)	7 (13.2)	
*Use of drugs last month (n = 122)* ^*c*^				0.579
No	119 (97.5)	68 (98.6)	51 (96.2)	
Yes	3 (2.5)	1 (1.4)	2 (3.8)	
*Frequency of alcohol consumption last month (n = 122)* ^*c*^				0.772
No	66 (54.1)	37 (53.6)	29 (54.8)	
1–5 days	44 (36.1)	24 (34.8)	20 (37.7)	
≥6 days	12 (9.8)	8 (11.6)	4 (7.5)	
*Doses of alcohol consumed on any occasion (n = 56)*^*c*^^,^^*d*^				0.007 ^b^
≤1 dose	12 (21.4)	4 (12.5)	8 (33.3)	
2–4 doses	22 (39.3)	10 (31.2)	12 (50)	
≥5 doses	22 (39.3)	18 (56.3)	4 (16.7)	
*Sexual intercourse (n = 122)*				0.120
No	39 (32)	26 (37.7)	13 (24.5)	
Yes	83 (68)	43 (62.3)	40 (75.5)	
*Number of sex partners (n = 83)*^*e*^				0.178
1 person	19 (22.9)	8 (18.6)	11 (27.5)	
2–3 people	18 (21.7)	7 (16.3)	11 (27.5)	
≥4 people	46 (55.4)	28 (65.1)	18 (45)	
*Age at first intercourse (n = 83)*^*c*^^,^^*e*^				0,467
≤13 years old	31 (37.8)	19 (44.2)	12 (30.8)	
14–15 years old	43 (52.4)	20 (46.5)	23 (59)	
≥16 years old	9 (9.8)	4 (9.3)	4 (10.2)	
*Preventive method against pregnancy and STDs (n = 79)*^*e*^				0.966
Yes	69 (87.3)	35 (87.5)	34 (87.2)	
No	10 (12.7)	5 (12.5)	5 (12.8)	

BMI, body mass index; STD, sexually transmitted disease.

^a^ Chi-square association test (χ²)

^b^ Significant association (p < 0.05)

^c^ Fisher’s Exact Test

^d^ Only students that consumption of time last month

^e^ Only students with active sex life.

## Discussion

The main results show that students, who were socioeconomically vulnerable, reported high levels of exposure to risk factors such as cigarette and alcohol consumption, risky sexual behavior and unhealthy diet. However, most of the students were physically active. Regarding the length of stay at the institution, students who had spent longer time at the institution presented with decreases in some unhealthy parameters, such as consumption of less alcohol, appropriate BMI and more nutritional knowledge when compared with those who spent less time at the institution.

The sample is composed mostly of male students. This is a characteristic of institutions of the Federal Education Network, which have basically offered agrarian courses in recent decades, and that attract predominantly male students for enrolment [12]. Although this reality is shifting with a greater variety of courses being offered and higher enrolment of women, the physical structure of the housing does not follow these changes and still offers more vacancies for men.

Fragile socioeconomic conditions are categorized according to the income level, a low level of education among legal guardians, and high prevalence of brown and black ethnicities/races. These data are consistent with the criteria used to select students who will benefit from housing at the institution and the inclusion of socioeconomic factors as a major health determinant worldwide [5]. Another factor that contributes to the students’ health risk is the distance from family, as the family is an important means of support in terms of protection and health promotion during adolescent development [13,15,36]. Studies have reported that a stronger familial bond is related to a later onset of sexual activity, reduced consumption of cigarettes, alcohol, and marijuana [5,15], and a better emotional state, welfare [10,36], and diet [15,16]. At universities, the existence of institutional housing and/or private residences is a common alternative intended to enable the student to stay at the educational institution in an attempt to encourage a better school performance [19]. However, this transition has been identified as an aggravating factor with respect to health-risk behaviors, as a consequence of the increased freedom caused by distance from the family [17]. It is noteworthy that little research has been conducted regarding residences at high school institutions, although institutional housing for high school students is a common situation [8] that is mainly associated with private schools [20]. However, we speculate that the students in our study underwent the same transition upon transferring to student housing, although this aggravating factor was encountered at an earlier age.

Therefore, the importance of assistance from the accommodating institution with respect to minimizing behaviors associated with socioeconomic vulnerability [1,37], enabling educational success and employment during adulthood, and breaking the vicious cycle of weaknesses by providing more favorable future prospects is evident [38]. Of note, adolescents in countries with higher socioeconomic differences (e.g., developing countries) are reported to have worse health conditions [38], an earlier onset of smoking [1] and alcohol and drug abuse, more risky sexual behavior [38], and inappropriate eating behaviors [6].

Our findings regarding substance use indicate that cigarettes and other drugs were used by a minority of the students; ideally, however, no consumption would be reported, especially given the ages of the studied adolescents. The percentage of students who currently smoked was 13.1%, similar to the prevalence rates reported in other studies [4,39–41] and greater than that reported by PENSE (6.1%) [27] and another Brazilian multicenter study of adolescents (5.7%) [42]. According to the World Health Organization (WHO), adolescents understand the act of smoking as an "adult behavior", and such behaviors are usually established in adolescence [43]. Cigarette smoking is a major cause of preventable death worldwide and is associated with the ingestion and consumption of other substances, such as drugs and alcohol [43], in addition to early sexual initiation [4,39,40].

Marijuana was the only reported drug (2.5%), and it is possible that the low reported drug consumption rate is due to a fear of confessing the consumption of illegal substances, even though the researchers ensured data confidentiality. PENSE reported similar 30-day consumption rates (3.1% of male and 2% of female subjects) [23]. In many countries worldwide, the use of drugs, especially marijuana, usually begins before 18 years of age [1,4,23,40] and thus comprises a public health problem with deep psychological and social consequences [39].

Regarding alcohol consumption by adolescents, studies have reported lower frequencies [4,23,39,44] than that observed in the present study, in which almost half of the students reported alcohol use during the last month. Another concern is the high consumption (≥5 doses) verified in the present study. We believe that distance from the family enhances the freedom of consumption, similar to the findings of studies of college students living at educational institutions [1,14–16].

As observed by other authors, the sexual behavior of students in this study is also indicative of this greater freedom associated with distance from the family [14–16]. Although most of the students used methods to prevent pregnancy and STDs and participated in educational activities about sexual and reproductive health, most reported multiple sexual partners; in addition, the age at first intercourse, another health risk factor, was lower than the ages reported in previous literature. Both risk behaviors are related to other problems and school performance. In this sense, a decline in the age at first intercourse and an increased rate of STDs have been perceived [4,45].

Most of the students had healthy diet scores of ≤30 points (below 70% of adequacy), indicating inadequate food intake. Previous reports have demonstrated that in adolescence, a diet rich in calories and sweetened beverages [46] and deficient in fibers, whole grains, vegetables, milk, dairy products, and unsaturated fat [47,48] is characteristic of a high consumption of unhealthy foods and insufficient consumption of healthy foods [4,47,49].

Regarding the new classification of food according to industrial processing [50], the consumption of natural or minimally processed foods has decreased within families, and the consumption of ultraprocessed foods has increased worldwide [51–53]. This change has resulted in many public health challenges, as these ultraprocessed foods are dense in energy, trans fats, sugars, sodium, and chemical additives [52,53] and have been associated with overweightness and cardiovascular disease in the Brazilian population [47,54], including adolescents [55]. Foods listed by PENSE as markers of a healthy diet are basically natural or minimally processed, whereas ultraprocessed foods are unhealthy markers. Although most students had a low healthy diet score when eating behavior was assessed as a whole, an analysis of individual food consumption revealed that students regularly consumed (≥5 times/week) of beans (89.4%), raw salad (60.6%), and milk and dairy products (51.6%) and infrequently consumed (≤5 times/week) fried snacks (96.7%), sausages (99.2%), sweet cookies (78.7%), sweets and desserts (73.8%), and soda (86.1%). Regarding the intake of other foods, our data corroborate the literature with respect to a high frequency of inadequate intake (i.e., low consumption of fruits and cooked vegetables). Therefore, although the students did not have ideal eating habits, as determined by the diet scores, adolescents who resided at the analyzed institution consumed a more adequate diet, according to the literature [47,56], than did other students of similar ages, as reported in a survey conducted in 43 countries in Europe and North America [4,46–49]. Another study of students residing at an educational institution indicated a reduced consumption of sweets in comparison to other young people [13].

To ensure a healthy diet, some universities with residential accommodations require students to purchase a meal plan in their first year, thus creating an incentive for them to eat meals on campus [14]. At the institution studied herein, four free daily meals are offered, and each includes meat, milk, eggs, and some vegetables, thus enhancing local sustainable production and the use of raw or minimally processed foods as recommended by the Food Guide for the Brazilian Population (*Guia Alimentar para a População Brasileira*) [56]. A healthy diet should be planned in the context of individual, cultural, social, economic, and sustainable aspects, and Brazil is among the first countries to include sustainability as a guideline in its food guide [57]. Although meals are mostly prepared in the institution's dining room, the students have access to unhealthy foods outside of the dining room; for example, these can be purchased at the cafeteria, brought from home, or exchanged among peers.

In terms of physical activity, most of the students were physically active. This finding contrasted the results of other studies of adolescents, which reported high percentages (≥60%) of insufficient physical activity in this age group [4,58]. A study of 105 countries worldwide found that 80% of adolescents were insufficiently active [59]. At the institution studied herein, the students have access to a court, soccer field, sand field, running track, swimming pool, and gymnasium, which help them to meet the physical activity recommendations. Also, teachers are available to practice some sports at counter-shift times, and students have access to all of these places on the weekend, along with assistants.

Given the presented context, educational institutions must effectively fulfill their role in the holistic education of students (i.e., physical, social, emotional, and spiritual development), especially when the institution is also the students' home [60]. The WHO notes that the existence of public policies and programs to promote the health and protect the rights of adolescents, who are at a vulnerable stage, is fundamental [61]. In this regard, international organizations such as the WHO and the United Nations Children's Fund (UNICEF) should recommend policies and prevention programs that countries can use to support and promote the health of adolescents [62]. A review of national public health policy documents from 109 countries found that 84% gave some type of attention to adolescents, with focuses on sexual and reproductive health, followed by tobacco, alcohol use, mental health and, less frequently, considerations of injuries, nutrition, and physical activity [61,62].

In Brazil, two major programs that target students are the School Health Program (*Programa Saúde na Escola*, PSE) and the National School Meal Program (*Programa Nacional de Alimentação Escolar*, PNAE). PSE represents a link between the school and the primary health care network that aims to promote health and a culture of peace by focusing on the prevention of health problems and working to ensure appropriate conditions for comprehensive education, among others [63]. PNAE aims to contribute to biopsychosocial growth and development, school performance, and healthy eating habits [64], which are rights guaranteed by the Statute of Children and Adolescents (*Estatuto da Criança e do Adolescente*, ECA). Despite the existence of these programs, our research highlights the fragility of their implementation at the institution in question.

In addition, the National Student Assistance Program (*Programa Nacional de Assistência Estudantil*, PNAES) aims to ensure the permanence of adolescents and young people in education in order to democratize permanence during academic life, minimize social and regional inequalities, and promote of social inclusion through education through subsidies targeting housing, food, and transportation [9]. However, this program does not incorporate a health-related theme or avoidance of risky behaviors, which should be included in all public policies and programs [7].

Regarding the time of residence at the public institution, students with a longer time of residence (Group 2) were older. This finding was expected, as most students begin residing at the institution during the first year of high school and remain in residence during the three years of school, even though they must compete for accommodation in any of the three years. The students also exhibited a higher prevalence of nutritional knowledge and BMI adequacy, in contrast to other studies that indicated decreases in these parameters with a longer institutional residence time, including a longitudinal study that monitored college students for 14 consecutive days during seven semesters [1,2,14]. Nutritional knowledge suggests that this subject might permeate the school curriculum, thus providing the recommended knowledge and skills related to the student [65], including nutritional education, which were sporadically performed. The appropriate BMI percentage might be related to the high participation in physically activity. In addition, students who had a longer length of stay at the institution consumed lower alcohol doses on any occasion. This result contradicts those of several surveys conducted among students that point to an increase in consumption during adolescence [40,66].

Given the presented perspective, despite the decreases in some unhealthy parameters being associated with a longer institutional stay, public policies and programs aim to improve student permanence and reduce socioeconomic vulnerabilities, but are weak with respect to health care and risky behaviors, when they should have health promotion and disease prevention as foundation. Possibly, the short duration of residence at the institution is not sufficient to change previous behaviors related to various factors, such as the family socioeconomic status. Therefore, it is important to provide conditions that will allow students to remain at the institution, as well as to develop dialogue mechanisms between public policy leaders and programs to ensure the well-being and health of this population. We suggest the development of activities from school admission (i.e., by conducting an individual assessment) to promote health education specific to the reality of the school. We also suggest the implementing of periodic actions, such as encouraging the students to engage in sports; increasing leisure and cultural options such as theater and dance; and promoting moments of family interaction inside the institution. These aspects could contribute to students making healthier decisions, thereby reducing the risk of behaviors negatively contributing to health.

In conclusion, high school students who reside at public educational institutions are exposed to health-risk behaviors that are aggravated by distance from their families. Nevertheless, a longer residence time is associated with some aspects such as better nutritional knowledge, an improved BMI and fewer consumed doses of alcohol.

## Study Limitations and Future Directions

This research is highly relevant because the amount of research related to the risk behaviors of high school students residing as educational public institutions is very limited. However, our results should be interpreted in light of certain restrictions. First, evaluations based on self-administered questionnaires have limitations such as biases toward socially desirable responses that might lead to an underestimation or overestimation of the problem. Second, we conducted the study at a single institution, which may cause bias. For this reason, additional studies conducted in other cultural and social settings (i.e., through longitudinal study) are needed because a cross-sectional study does not provide information about the nature of the risk behaviors. Third, at the assessed institution there are some peculiarities, such as an offering of several spaces for the students to play different sports; this may have contributed to our results, and because of this, our findings should be extrapolated with caution. Future research should investigate aspects such as school environment and social and public health policy and their influence on health-risk behaviors.

## Supporting Information

S1 Dataset Health-risk behaviors: Datasets of participants. (XLS)
